# The Effect of Beta Adrenoreceptor Blockers on Viability and Cell Colony Formation of Non-Small Cell Lung Cancer Cell Lines A549 and H1299

**DOI:** 10.3390/molecules27061938

**Published:** 2022-03-17

**Authors:** Marina Sidorova, Vilma Petrikaitė

**Affiliations:** Laboratory of Drug Targets Histopathology, Lithuanian University of Health Sciences, A. Mickeviciaus g. 9, LT-44307 Kaunas, Lithuania; marina.sidorova348@gmail.com

**Keywords:** beta adrenoblocker, anticancer, non-small cell lung cancer, clonogenic, apoptosis, necrosis

## Abstract

Beta adrenoblockers are a large class of drugs used to treat cardiovascular diseases, migraines, glaucoma and hyperthyroidism. Over the last couple of decades, the anticancer effects of these compounds have been extensively studied. However, the exact mechanism is still not known, and more detailed studies are required. The aim of our study was to evaluate the anticancer activity of beta adrenoblockers in non-small cell lung cancer cell lines A549 and H1299. In order to find the relationship with their selectivity to beta adrenoreceptors, selective (atenolol, betaxolol, esmolol, metoprolol) and non-selective (pindolol, propranolol and timolol) beta blockers were tested. The effect on cell viability was evaluated by MTT assay, and the activity on cell ability to form colonies was tested by clonogenic assay. The type of cell death was evaluated by cell double staining with Hoechst 33342 and Propidium iodide. The most active adrenoblockers against both tested cancer cell lines were propranolol and betaxolol. They completely inhibited lung cancer cell colony formation at 90% of the EC_50_ (half-maximal effective concentration) value. Most tested compounds induced cell death through apoptosis and necrosis. There was no correlation established between beta adrenoblocker anticancer activity and their selectivity to beta adrenoreceptors.

## 1. Introduction

Lung cancer is the most common type of cancer and a leading cause of death worldwide, accounting for an estimated 9.6 million deaths in 2018 [1]. Despite progress in diagnostics and treatment, lung cancer therapy remains problematic. Resistance to drug treatment is the main reason for the decreasing effectiveness of therapy [2], and the five-year survival rate is less than 18% [3].

The catecholamines norepinephrine and epinephrine, also called noradrenaline and adrenaline, are neurotransmitters that tare simultaneously released from the sympathetic nervous system and adrenal gland as a response to physiological and psychological stress, otherwise called flight-or-fight response. They regulate the activity of organs and cells related to stimulation of the sympathetic nerve system. According to scientists, the elevated concentration of catecholamines promotes growth of lung adenocarcinoma micro metastasis [4]. Conversely, beta adrenergic receptor antagonists (beta adrenoblockers) stop the binding of norepinephrine and epinephrine by occupying the active site of the beta adrenoceptors, thereby decreasing their stimulation and risk of the growth of cancer.

Beta adrenoblockers are a large class of drugs mainly used to manage cardiovascular diseases, migraines, glaucoma and hyperthyroidism. Over the last couples of decades, the anticancer effects of these compounds have been extensively studied. The first evidence about beta adrenoreceptor involvement in lung cancer development occurred in 1989 [5]. According to recent studies, beta blockers also possess anticancer activity in pancreatic, breast, colorectal, prostate and ovarian cancer [2,3,6]. The researchers concluded that stimulation of beta adrenoreceptors by catecholamines leads to an increase in the extracellular concentration of the cyclic adenosine monophosphate, which promotes the proliferation of cancer cells [7,8]. These neuromediators promote resistance of cancer cells and tumor formation and growth by inhibiting the activity of the immune system, which involves a decrease in the amount and activity of lymphocytes and natural killer cells [9]. In animal models, the antagonistic effect of non-selective beta adrenoreceptors blockers on beta-2 adrenoreceptors reactivated functioning of lymphocytes but did not improve survival outcomes [10]. However, beta adrenoblockers in combination with COX-2 inhibitors improved survival rates of mice [11]. It was proven that through the activation of beta adrenoreceptors, COX-2 becomes active, and therefore cancer cell growth and invasion is promoted through the arachidonic acid pathway [12,13]. Despite the scientific evidence that beta blockers have been shown to reduce the proliferation, migration, invasiveness, and angiogenesis of cancer cells and tumor immune response [10,14,15], the exact antitumor mechanism of action remains unclear.

According to the results of clinical trials, beta adrenoblockers increase the survival rate of patients suffering from breast, prostate, ovarian, colorectal, skin and lung cancer. In recent years, new evidence has shown that overall survival of patients, who received beta adrenoblockers combined with radiotherapy, increased by 22% compared to the control group [16]. Despite evidence of the positive effect of beta adrenoblockers on patients’ survival, some data show that the intake of beta adrenoblockers and other medicines affecting the metabolism of catecholamines is associated with an increased risk of the development of cancer and higher mortality [17,18]. It has been shown that beta adrenoblockers may sensitize the non-small cell lung cancer (NSCLC) cells to chemoradiation and possibly decrease distant metastases [19]. The expression of beta-1 and beta-2 receptors was determined in NSCLC tissues by Coelho et al., especially in adenocarcinoma samples [20]. A number of preclinical and clinical evidence related to the beta-adrenergic signaling in lung cancer has been reviewed by Nilsson et al. [21], supporting the idea of repurposing beta adrenoblockers for the application of NSCLC treatment.

Although the anticancer activity of beta adrenoblockers has been observed for almost two decades, no unified anticancer mechanism of action was discovered. Considering the problem and prevalence of lung cancer treatment, we decided to investigate the anticancer activity of beta adrenoblockers in the NSCLC lines A549 and H1299. These cell lines are derived from lung adenocarcinomas, but they represent different cell types. A549 cell line is a type II alveolar epithelium of characteristic morphology and functions [22,23], and H1299 is a small airway (bronchiolar) epithelial cell line. Also, these cell lines are characterized by a different sensitivity to anticancer drugs [24]. A549 and H1299 cells express both beta-1 and beta-2 adrenergic receptors [20,25], beta-2 receptors being predominant in the A549 cell line [26].

In this work, the beta adrenoblockers’ effect on cell viability, clonogenicity and the type of cell death was investigated. As the model compounds for our study, we chose the first-generation beta adrenoblockers acting as antagonists on both beta-1 and beta-2 adrenoreceptors (non-selective compounds pindolol, propranolol and timolol). Also, the second-generation beta adrenoblockers, which bind stronger to the beta-1 receptors, were selected (atenolol, betaxolol, esmolol, metoprolol). Pindolol also has partial agonist activity [27,28]. We aimed to explore possible relationship between their anticancer activity and selectivity to beta adrenoreceptors.

## 2. Results

### 2.1. Beta Adrenoblockers Reduce the Viability of NSCLC Cells

**Figure 1:**
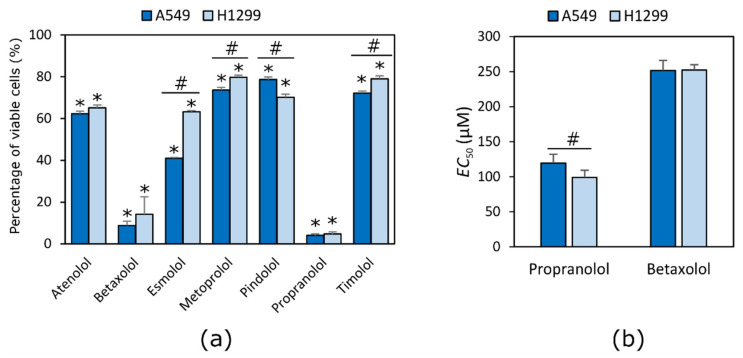
Effect of beta adrenoblockers on NCLSC cell viability. (**a**) effect of all tested compounds 500 μM concentration on A549 and H1299 cell viability; (**b**) EC_50_ values of propranolol and betaxolol. * *p* < 0.05, compared to control; # *p* < 0.05, compared activity between cancer cell lines.

All tested compounds reduced NSCLC cell viability at the highest used concentration of 500 µM. Propranolol and betaxolol were the most active compounds in both cell lines (Figure 1a). After evaluation of EC_50_ values of both most active compounds, propranolol showed a stronger effect on viability of the H1299 cell line, while betaxolol acted in the same way in both cell lines (*p* < 0.05) (Figure 1b).

Propranolol possessed the highest antiproliferative activity (EC_50_ values were 119.3 ± 12.7 µM and 98.8 ± 10.3 µM in A549 and H1299 cell lines, respectively). Betaxolol activity was about twice lower compared to propranolol (EC_50_ values were 251.3 ± 14.6 µM and 252.2 ± 7.6 µM in A549 and H1299 cell lines, respectively).

### 2.2. Beta Adrenoblockers Inhibit Growth of Cell Colonies in Concentration-Dependent Way

**Figure 2:**
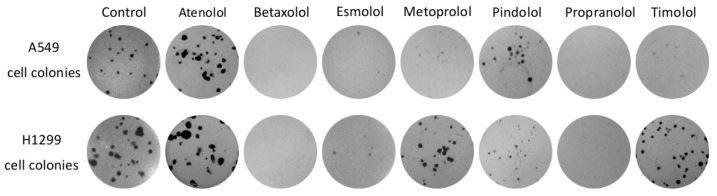
A549 and H1299 cell colonies after incubation with 90% of EC_50_ concentrations of beta adrenoblockers. Each figure represents the whole area of the well bottom from the 12-well plate.

**Figure 3:**
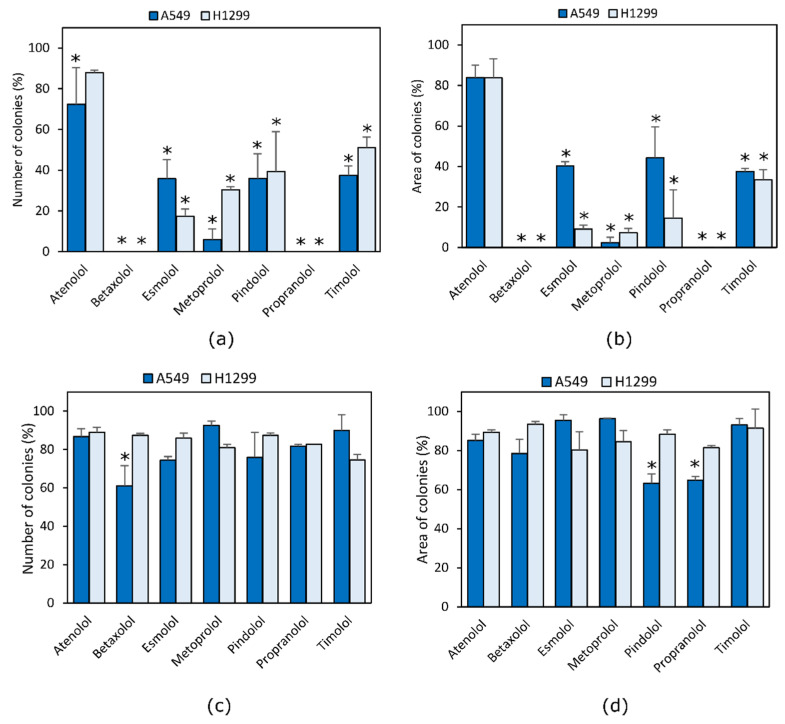
Effect of beta adrenoblockers on NCLSC cell colony formation ability. Comparison of compound effect of 90% of their EC_50_ value on (**a**) cell colony number and (**b**) area of colonies; and compound effect of 10% of EC_50_ value on (**c**) cell colony number and (**d**) area of colonies. * *p* < 0.05, compared to control.

The tested beta adrenoblockers showed a different effect on NCLSC cell colony formation (Figure 2). Propranolol and betaxolol at a concentration of 90% of EC_50_ value completely suppressed colony formation ability in both cell lines (*p* < 0.05) (Figure 3a,b). All compounds except for atenolol at the higher concentration inhibited growth of cell colonies.

Slightly weaker than propranolol and betaxolol, the number and area of A549 cell colonies was reduced by metoprolol at the higher used concentration in this study. The non-selective beta blockers timolol and pindolol and the selective beta blocker esmolol were found to possess a similar activity between them. Also, these non-selective beta blockers were less active compared to propranolol and betaxolol. The lowest inhibition of A549 cell colony formation ability was established for the selective beta blocker atenolol.

Similar trends of the effect of beta blockers on the H1299 cell line have been identified in the study. All compounds except atenolol had a statistically significant reduction in the number of colonies and the area occupied by these cells (*p* < 0.05). The most active compounds were propranolol and betaxolol, while esmolol and metoprolol showed slightly lower activity (*p* < 0.05).

Only betaxolol at a concentration of 10% of EC_50_ value inhibited the growth of A549 cells colonies, while pindolol and propranolol also decreased the size of colonies compared to the control group (*p* < 0.05) (Figure 3c,d). None of the compounds at lower concentrations had an effect on the growth and size of the H1299 cell colonies (*p* > 0.05).

### 2.3. Beta Adrenoblockers Mainly Cause Apoptosis

**Figure 4:**
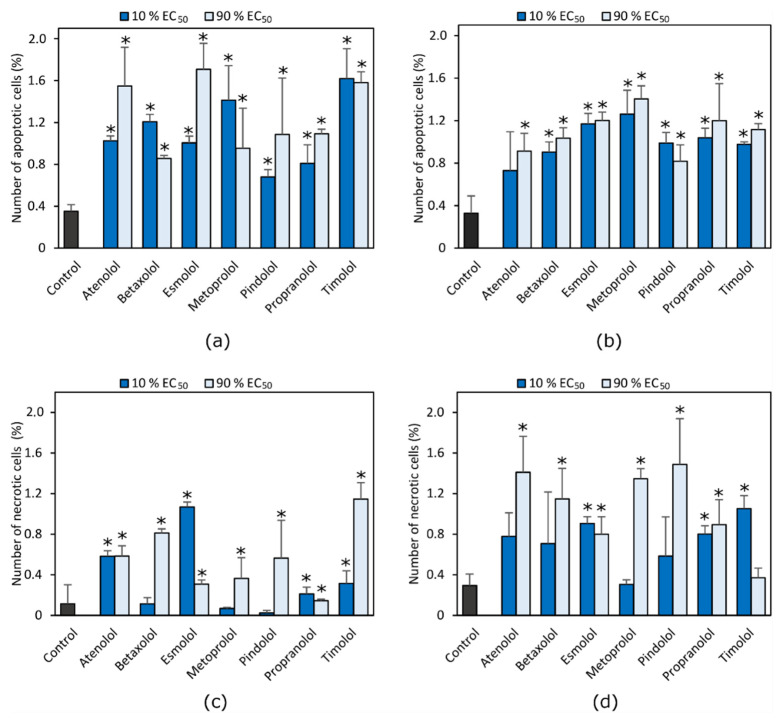
The effect of beta adrenoblockers on NCLSC cell death type. Number of apoptotic cells in (**a**) A549; (**b**) H1299 cancer cell lines and number of necrotic cells in (**c**) A549 and (**d**) H1299 cell lines. * *p* < 0.05, compared to the control.

Most tested compounds induced cell death through apoptosis and necrosis. In A549 cell lines, apoptosis was mainly induced, while in H1299 cell line compounds induced both apoptosis and necrosis (Figure 4).

All the tested compounds induced apoptosis in the A549 cell line even at concentration of 10% of the calculated EC_50_ value (*p* < 0.05) (Figure 4a). No statistically significant difference was found between the apoptotic effect of beta adrenoblockers at the concentration of 10 and 90% of calculated EC_50_ value on A549 cells (*p* > 0.05). Only atenolol at a lower concentration did not induce apoptosis in the H1299 cell line (*p* > 0.05) (Figure 4b).

No statistically significant difference was found between the beta adrenoblockers’ effect on cell apoptosis between cell lines.

Beta adrenoblockers mainly induced necrosis in H1299, but not in the A549 cell line (Figure 4c). All of the tested compounds with the exception of timolol at a higher concentration induced necrosis in the H1299 cell line (*p* < 0.05) (Figure 4d). Metoprolol, pindolol and betaxolol did not cause necrosis of A549 cells. Only esmolol, propranolol and timolol at a lower concentration induced necrosis in the H1299 cell line (*p* < 0.05).

## 3. Discussion

The effect of beta adrenoblockers on cell viability is a common subject of different scientific studies. Propranolol after 72 h of incubation inhibited cell viability of lung cancer A375 and melanoma P8 cell lines at concentrations 77.30 and 60.30 μM, respectively [18]. Similar results were obtained in myeloma U266 cell line [29]. The difference between the calculated EC_50_ values can be explained by the fact that the expression of receptors varies between different types of cell lines. In general, it is thought that non-selective beta adrenoblockers have a stronger effect on cell viability than that of beta-1 selective compounds [30]. Atenolol was from 7 to 50 times less active than propranolol in breast MCF-7, colorectal HT-29 and hepatocellular HepG2 cell lines. Similar results were achieved in this study. Atenolol was six times less active than propranolol. The amount of living cells after exposure to atenolol was 62.26% in H1299 and 65.12% in the A549 cell line, compared to 4.13% and 4.73% for propranolol, respectively. Propranolol is a non-selective beta adrenoblocker whilst atenolol is selective. Moreover, propranolol possesses membrane stabilizing activity. However, this tendency was not noticed in examining the activity of the other five substances used in the experiment. One of the most active compounds—betaxolol—is selective and propranolol is a non-selective beta adrenoblocker.

We found that the antiproliferative activity of beta adrenoblockers is not correlating with their selectivity to the receptors and might be dependent on the compound lipophilicity and membrane stabilizing activity. Beta-2 adrenoreceptors in lung adenocarcinoma are responsible for lymphatic permeation and vascular invasion [31]. However, the expression of beta-2 adrenoreceptors in lung adenocarcinoma is not associated with worse survival outcomes in patients. In this study, only one of the non-selective beta adrenoblockers, propranolol, inhibited cell viability at a concentration less than 500 µM. Betaxolol and propranolol possess the same selectivity to beta-1 adrenoceptors. However, the non-selective compound pindolol with the strongest beta-1 antagonistic activity of all the tested compounds was the least active compound in the A549 cell line, but one of the most active compounds in the H1299 cell line. The selective adrenoblockers esmolol and atenolol also were one of the most active compounds in H1299 cells, which might be proof that the expression of adrenoreceptors varies in cell lines themselves and that the selectivity of compounds is not the most important feature in predicting the anticancer activity of a substance. Zhang and the group suggested that the activated k-ras gene mutation in cell lines might be responsible for the lower activity of beta-2 adrenoreceptor blockers [32]. This explains why propranolol was more active in the H1299 cell line (*p* < 0.05), while betaxolol activity was the same in both cell lines (*p* > 0.05). However, both NSCLC cell lines, A549 and H1299, possess the K-Ras gene mutation that is thought to be responsible for lower sensitivity to non-selective beta adrenoblockers [32]. In addition, the mutation varies between the cell lines [33].

The effect of beta adrenoblockers on colony formation is not a common subject of scientific research. Min discovered that propranolol and atenolol at 10 µM concentrations suppresses the growth and ability of A549 and H0CC-15 cells, treated by NNK, to form colonies [34]. In this study, a 12 µM concentration of propranolol reduced the size of A549 cell colonies, but atenolol, even at a 450 µM concentration, did not have a statistically significant effect on colony growth. The deviation from expected results could be explained by the differences in laboratory techniques. There is also evidence that propranolol in combination with radiotherapy and sumatinib reduces the clonogenicity of stomach cancer and melanoma [18,35].

Zhang concluded that a 100 μM concentration of metoprolol does not cause apoptosis in pancreatic cell lines [32]. In this study, metoprolol, even at 50 μM concentration, induced apoptosis in the A549 and H1299 cell lines. The results of experiments may differ due to the variation of expression of beta adrenoreceptors in cell lines and the mechanism of action of drugs through metabolic pathways.

In another study, propranolol at 50 μM concentration did not cause apoptosis of gastric adenocarcinoma in the BGC-823 and SGC-7901cell lines, but in combination with radiotherapy after 48 h incubation it induced apoptosis, clonogenic survivability and cell viability [35]. In our experiment, propranolol induced apoptosis at a 12 μM concentration. However, cells were incubated with solutions of compounds for 72 h. The ability of beta adrenoblockers to cause apoptosis may be time dependent.

In order to evaluate impact of beta adrenergic receptors on the type of cell death, the effect of the beta-2 selective adrenoblocker butoxamine, non-selective propranolol and beta-1 selective metoprolol were used to induce apoptosis in a PC-2 pancreatic cancer cell line [36]. The apoptosis rate was the lowest after treatment with metoprolol, and the highest after treatment with butoxamine. According to the results of this study, it can be stated that the apoptotic effect of beta adrenoblockers is mainly dependent on selectivity to beta-2 adrenoreceptors. It is worth noting that Zhang and others used only single compounds that possess specific selectivity to a certain type of receptors. In our study, lung cancer cell lines were treated with several different compounds possessing different selectivity towards beta adrenoreceptors, but no statistically significant differences between their effects were noticed. Moreover, different concentrations of compounds were used. It may be presumed that the selectivity of beta adrenoblockers is important for anticancer activity in some specific cell lines, but not all of them in general.

## 4. Materials and Methods

### 4.1. Chemicals and Materials

Atenolol (99% pure), betaxolol (96% pure), esmolol (98% pure), timolol (99% pure) and pindolol (99% pure) were purchased from Abcam (Cambridge, UK), metoprolol (98% pure) was purchased from Alfa Aesar (Ward Hill, MA, USA), and propranolol (99% pure) was purchased from Acros Organic (Morris Plains, NJ, USA). All tested compounds were dissolved in dimethylsulfoxide (DMSO, ≥99%, Ph. Eur.) which was obtained from Sigma-Aldrich Co. (St. Louis, MO, USA).

TrypLE Express, Dulbecco’s modified Eagle high glucose medium (DMEM GlutaMAX), fetal bovine serum (FBS), penicillin/streptomycin solution (10,000 IU/mL), and phosphate buffered saline (PBS, pH = 7.4) were purchased from Gibco (Carlsbad, CA, USA). The aqueous 16% paraformaldehyde solution (PFA), the Hoechst 33342 (1 mg/mL) solution, and the Propidium iodide (1 mg/mL) solution were obtained from Thermo Fisher Scientific (Heysham, UK).

3-(4,5-Dimethylthiazol-2-yl)-2,5-diphenyltetrazolium bromide (MTT, ≥97%) and crystal violet (≥90%) were purchased from Sigma-Aldrich Co. (St. Louis, MO, USA). Ethanol (96.6%) was obtained from Stumbras, LLC (Kaunas, Lithuania).

All cell culture plastic ware was purchased from Thermo Fisher Scientific, Corning (Phoenix, AZ, USA) and Techno Plastic Products (Trasadingen, Switzerland).

### 4.2. Cell Culture

Human NSCLC cell lines A549 and H1299 were obtained from the American Type Culture Collection (ATCC, Manassas, VA, USA). Both cell lines were cultured in Dulbecco’s Modified Eagle’s Medium GlutaMAX (Gibco, Carlsbad, CA, USA), supplemented with 10% FBS and 1% antibiotics. Cells were incubated at 37 °C temperature in a humidified atmosphere containing 5% CO_2_. All cell cultures routinely were grown to 70% confluence and trypsinized with 0.125% TrypLE™ Express solution (Gibco, Carlsbad, CA, USA) before passage. They were used until passage 20.

### 4.3. Cell Viability Assay

Cell viability was evaluated by MTT assay, as described elsewhere [37]. Briefly, A549 and H1299 cells were seeded in a 96-well plate at a concentration 5000 cells/well and incubated overnight. After 24 h, cells were affected by different concentrations of beta adrenoblockers. The medium without cells served as a positive control, and the cells treated with medium containing 0.5% DMSO was used as a negative control.

After 72 h, 20 µL of MTT 0.5 mg/mL solution was added into each well of a 96-well plate, and cells were incubated at 37 °C for 3 h. Next, the supernatant was removed and the formed formazan crystals were dissolved in 100 µL of DMSO. The absorbance was measured at 570 nm and 630 nm reference wavelengths using a multi-detection microplate reader. Experiments were repeated three times independently and the results were presented as means ± SD.

Applying Hill fit to compound dose—cell metabolic activity (absorbance) curves, the half maximal effective concentration (EC_50_) values, reducing cell viability by 50%, were calculated.

### 4.4. Cell Colony Formation Assay

The compound effect on cell colony formation was tested by clonogenic assay as described elsewhere [38]. Briefly, 1000 of A549 and H1299 cells in a volume of 1 mL were seeded in a 12-well and then were treated with 100 µL of 10 or 90% of EC50 values of adrenoblockers. The medium containing 0.5% of DMSO served as a negative control. H1299 cells were incubated for eight days, and A549 was incubated for 12 days at 37 °C in an atmosphere containing 5% CO_2_. The colonies were then rinsed twice with PBS and fixed with 4% paraformaldehyde solution in PBS for 15 min. Colonies were stained with a 0.1% aqueous crystal violet solution for 15 min and washed twice with sterile deionized water. Pictures were taken using a G:BOX gel documentation system (Syngene International Ltd., Bengaluru, India) and analysed using Genesys software (Syngene International Ltd.). The number and percentage area of colonies were calculated.

### 4.5. Evaluation of Type of Cell Death

Lung cancer cells were seeded in 24-well plates at a concentration 15,000 cells/well and incubated for 24 h at 37 °C in an atmosphere containing 5% CO_2_. Next, either 10 or 90% of EC_50_ values of adrenoblockers were added to the wells. After 72 h, 3 µL of aqueous solution of Hoechst 33342 (1 mg/mL) and 1 µL of aqueous solution of Propidium iodide (1 mg/mL) were added to each well. After 10 min, images of cells were taken by an inverted fluorescent microscope (Olympus IX73, Shinjuku, Japan). Apoptotic and necrotic cells were counted, and the percentage number of cells was calculated.

Hoescht 33342 is a cell permeable dye and binds to the DNA in cells. Therefore, the stained nuclei of vital cells emit blue-cyan fluorescent light and are visible as blue colored. Apoptotic cells display condensed DNA and fragmented nuclei in blue. Propidium iodide is not cell permeable and binds to the double-stranded DNA of cells where the plasma membrane has been compromised. Non-viable, necrotic cells are seen as red colored. Propidium iodide does not stain live or early apoptotic cells due to the presence of an intact plasma membrane [39].

### 4.6. Statistical Analysis

A statistical analysis was performed using Microsoft Office Excel 2007 software (Microsoft Corporation, Redmond, WA, USA), evaluating an average and standard deviation of at least three measurements. A Student’s *t*-test was used and *p*-values were calculated. A value of *p* < 0.05 was considered as the level of significance.

## 5. Conclusions

Our results show that both selective and non-selective beta adrenoblockers, especially betaxolol and propranolol, reduce the viability of the NSCLC cell lines H1299 and A549. Propranolol, which is a non-selective beta adrenoblocker, showed the strongest effect on H1299 cell viability, while the selective agent betaxolol possessed a similar activity against both tested cell lines. Betaxolol activity was about twice lower compared to propranolol (EC_50_ values were 251.3 ± 14.6 µM and 252.2 ± 7.6 µM in A549 and H1299 cell lines, respectively). Therefore, it could be concluded that the cytotoxicity of beta adrenoblockers against the tested lung cancer cell lines is not dependent on their selectivity on beta adrenoreceptors.

Overall, it has been established that betablockers inhibit the formation of cell colonies and induce apoptosis and necrosis. No statistically significant difference was found between the beta adrenoblocker effect on cell apoptosis in both NSCLC cell lines. In A549 cell lines, apoptosis was mainly induced, while the H1299 cell line compounds induced both apoptosis and necrosis.

The anticancer activity of the tested beta adrenoblockers is not related to the selectivity to beta adrenoreceptors. Therefore propranolol and betaxolol showed the strongest anticancer activity in vitro, and both compounds are worthy of further investigation and could be considered as therapeutic alternatives with regard to solving chemoresistance.

## Data Availability

The datasets used and/or analyzed during the current study are available from the corresponding author on reasonable request.

## Sample Availability

Compounds used in this study are commercially available.
